# *Vernonia kotschyana* Roots: Therapeutic Potential via Antioxidant Activity

**DOI:** 10.3390/molecules191119114

**Published:** 2014-11-19

**Authors:** Alexandru Vasincu, Berit S. Paulsen, Drissa Diallo, Ioana Vasincu, Ana C. Aprotosoaie, Veronica Bild, Christiana Charalambous, Andreas I. Constantinou, Anca Miron, Cristina M. Gavrilescu

**Affiliations:** 1 Department of Pharmaceutical Sciences II, Faculty of Pharmacy, University of Medicine and Pharmacy Grigore T. Popa-Iasi, Universitatii Str. 16, 700115 Iasi, Romania; E-Mails: alexandru.vasincu@umfiasi.ro (A.V.); ana.aprotosoaie@umfiasi.ro (A.C.A.); veronica.bild@umfiasi.ro (V.B.); 2 Section of Pharmaceutical Chemistry, School of Pharmacy, University of Oslo, P.O. Box 1068 Blindern, N-0316 Oslo, Norway; E-Mail: b.s.paulsen@farmasi.uio.no; 3 Department of Traditional Medicine, University of Bamako, PB 1746 Bamako, Mali; E-Mail: dri.diallo@yahoo.fr; 4 Department of Pharmaceutical Sciences I, Faculty of Pharmacy, University of Medicine and Pharmacy Grigore T. Popa-Iasi, Universitatii Str. 16, 700115 Iasi, Romania; E-Mail: ioana-mirela.vasincu@umfiasi.ro; 5 Department of Biological Sciences, University of Cyprus, Kallipoleos 75, 1678 Nicosia, Cyprus; E-Mails: ccharala@hotmail.com (C.C.); andreasc@ucy.ac.cy (A.I.C.); 6 Department Medical I, Faculty of Medicine, University of Medicine and Pharmacy Grigore T. Popa-Iasi, Universitatii Str. 16, 700115 Iasi, Romania

**Keywords:** *Vernonia kotschyana*, ethyl acetate extract, free radical scavenging activity, lipid peroxidation inhibition, FT-IR spectroscopy, phenolic content

## Abstract

The roots of* Vernonia kotschyana* Sch. Bip. ex Walp. (Asteraceae) are used in Malian traditional medicine in the treatment of gastroduodenal ulcers and gastritis. Since oxidative stress is involved in gastric ulceration, the aim of this study was to screen the root extracts for their* in vitro* antioxidant activity and phenolic content. The roots were extracted successively with chloroform, ethyl acetate, ethanol and water. The antioxidant activity of root extracts was evaluated in both cell-free and cell-based assays. Their chemical characterization was performed by Fourier transform infrared spectroscopy (FT-IR) whereas the total phenolic content was determined by the Folin-Ciocalteu method. The ethyl acetate extract displayed the highest phenolic content and was found to be the most active in the free radical scavenging and lipid peroxidation inhibition assays; it also showed a high antioxidant activity in MCF-12F cells. This study suggests a potential use of the ethyl acetate extract of *Vernonia kotschyana* not only as an antioxidant agent in gastroduodenal ulcers and gastritis, but also in other disorders characterized by high levels of oxidative stress.

## 1. Introduction

Gastric ulcers have been treated with herbal drugs for centuries. Ethnopharmacological surveys reveal numerous plant species used in the treatment of gastric ulcer. For many of them, pharmacological studies have elucidated the mechanisms of antiulcer activity thus justifying the traditional use. Anti-*Helicobacter pylori*, antisecretory, cytoprotective and antioxidant effects are mainly involved in the antiulcer activity of plant extracts [1,2].

Reactive oxygen species (ROS) and, to a lesser extent, reactive nitrogen species (RNS) play a critical role in gastric ulceration. *Helicobacter pylori*, the main causative factor of gastric ulcers and gastritis, recruits neutrophils and macrophages to the site of infection where they release high levels of ROS (*oxidative burst*). First, superoxide anion radical is produced by membrane-associated nicotinamide adenine dinucleotide phosphate oxidase (NADPH oxidase). Superoxide anion radical can be further converted into hydrogen peroxide both enzymatically (via superoxide dismutases) and non-enzymatically. In the presence of transition metal ions (ferrous and cuprous ions), superoxide anion radical and hydrogen peroxide interact and form the highly reactive hydroxyl radical. Neutrophil myeloperoxidase generates hypochlorous acid from hydrogen peroxide and chloride anions; hypochlorous acid is a precursor of chloramines, oxidant and cytotoxic species. This overproduction of ROS is the major cause of gastric mucosal damage. In addition, *Helicobacter pylori* induces the expression of inducible nitric oxide synthase (iNOS) in the gastric mucosa thus promoting the generation of nitric oxide and peroxynitrite anion. The latter, produced by the reaction between nitric oxide and superoxide anion radical, is highly cytotoxic [3]. Some of these reactive species (hydroxyl radical, peroxynitrite anion) mediate lipid peroxidation processes with subsequent cell lysis and generation of cytotoxic products such as malondialdehyde and 4-hydroxynonenal [4,5]. Oxidative stress is also involved in the gastric ulcerations caused by ethanol, nonsteroidal anti-inflammatory drugs (NSAIDS) and cold restraint stress [6,7]. Many human studies support the involvement of oxidative stress in gastric ulcerations. Low levels of glutathione, high levels of malondialdehyde, changes in gastric mucosal superoxide dismutase and glutathione peroxidase activities and high levels of nitric oxide in gastric juice have been reported in patients with gastric ulcers. In addition, a decrease in serum levels of endogenous antioxidants (glutathione, vitamins E and C) and an increase in serum level of malondialdehyde have been detected [8,9].

The antiulcer activity of many plant extracts is largely related to their antioxidant potential. Besides its anti-*Helicobacter pylori* and cytoprotective effects, the aqueous extract of *Zingiber officinale* rhizome increased the level of reduced glutathione, decreased lipid peroxidation and normalized the activity of antioxidant enzymes (catalase, superoxide dismutase, glutathione peroxidase) in the gastric mucosa and serum in swim/ethanol stress-induced ulcer models [10]. *Parkia speciosa* ethanol leaf extract significantly decreased lipid peroxidation and increased the levels of glutathione and superoxide dismutase in the gastric mucosa in rats with ethanol-induced gastric ulcers. In addition, the extract showed the ability to upregulate the heat-shock protein 70 (HSP70) and downregulate the pro-apoptotic protein BAX, the former being involved in cytoprotection against different stresses, including oxidative stress [11]. The ethanol leaf extract of other *Parkia* species, *P. platycephala*, protected against ethanol-induced gastric lesions by different mechanisms including activation of catalase [12]. The gastroprotective effects of *Opuntia ficus indica f. inermis* flowers extract against ethanol-induced gastric mucosal damage were reported to be based on its antioxidant potential (inhibition of lipid peroxidation, protein oxidation, DNA fragmentation and myeloperoxidase activity, the latter being an index of neutrophil infiltration, restoration of the activities of superoxide dismutase, catalase and glutathione peroxidase) [13]. In different experimental models (water immersion stress, cold restraint stress, ethanol, aspirin, pylorus ligation-induced gastric ulcers in rats), extracts from strawberry cultivars (*Fragaria × ananassa*), *Curcuma longa*, *Mentha piperita* and *Moringa oleifera* were all reported to reduce gastric injury by decreasing lipid peroxidation and normalizing the activity of antioxidant enzymes [14,15,16].

The *Vernonia* genus (Asteraceae) includes around one thousand species distributed worldwide, mainly in the tropical regions. Many *Vernonia* species are used as foods (*V. amygdalina*, *V. colorata*) or herbal medicines (*V. amygdalina*, *V. condensata*, *V. conferta*, *V. cinerea*, *V. guineensis*), and several species have industrial uses (*V. galamensis*) [17]. The roots of* Vernonia kotschyana* Sch. Bip. ex Walp. syn. *Baccharoides adoensis* var. *kotschyana* (Sch. Bip. ex Walp.) are used in Malian traditional medicine in the treatment of gastrointestinal disorders (gastroduodenal ulcers, gastritis, indigestion) and wounds [18,19]. A Malian improved traditional medicine, *Gastrosedal*, consisting of the dried powdered roots, is recommended to be administered as a decoction in gastroduodenal ulcers and gastritis; a small clinical trial demonstrated its efficacy in gastric ulcer patients [18]. To our knowledge, only the biological effects of the root aqueous extracts and some of their constituents (polysaccharide fractions) have been investigated. The macerate was reported to reduce the number and severity of ethanol-induced gastric lesions in rats (hyperemia and thickened lesions of the mucosa) [20]. Acidic polysaccharide fractions containing type II arabinogalactan structures and inulin-rich fractions were isolated from the 50 °C and 100 °C water extracts. Acidic polysaccharides showed complement fixation and induction of B-cell proliferation effects; these immunomodulatory activities may, in part, explain an anti-inflammatory and tissue repair potential. Inulin-rich fractions showed no immunomodulating activities (complement fixation, macrophage activation) but reduced 0.3 M HCl-60% ethanol-induced gastric lesions in mice [18,19]. A recent study showed that the 100 °C crude water extract and two pectic polysaccharides inhibited *Helicobacter pylori* adhesion to gastric adenocarcinoma epithelial cells; the most potent activity (approximately 30% decrease in *Helicobacter pylori* adherence) was detected for the arabinose-rich pectic polysaccharide [21]. As the roots of *V. kotschyana* are used to produce *Gastrosedal*, the wild populations have diminished considerably. Therefore, the plant is now cultivated in several areas in Mali. No significant differences were detected in the chemical composition, complement fixation and macrophages stimulation between polysaccharides from cultivated and wild *V. kotschyana* roots suggesting that the cultivated roots can replace wild ones in producing *Gastrosedal* and other herbal medicines [22].

Despite the fact that oxidative stress plays an important role in gastric ulceration, the antioxidant potential of *V. kotschyana* roots has not been investigated yet. In this respect, our goal was to evaluate the antioxidant potential of root extracts in relation to antiulcer activity and other pharmacological activities partially mediated by antioxidant effects. The present work reports data on the antioxidant activity of different extracts of *V. kotschyana* roots assessed by several* in vitro* assays.

## 2. Results and Discussion

Successive extractions of *V. kotschyana* roots with solvents of increasing polarity led to four extracts: chloroform (V-C), ethyl acetate (V-EA), ethanol (V-E) and aqueous (V-A) extracts; the yields were found to be 1.1%, 2.08%, 4.82% and 6.95%, respectively.

### 2.1. Free Radical Scavenging Activity

**Figure 1:**
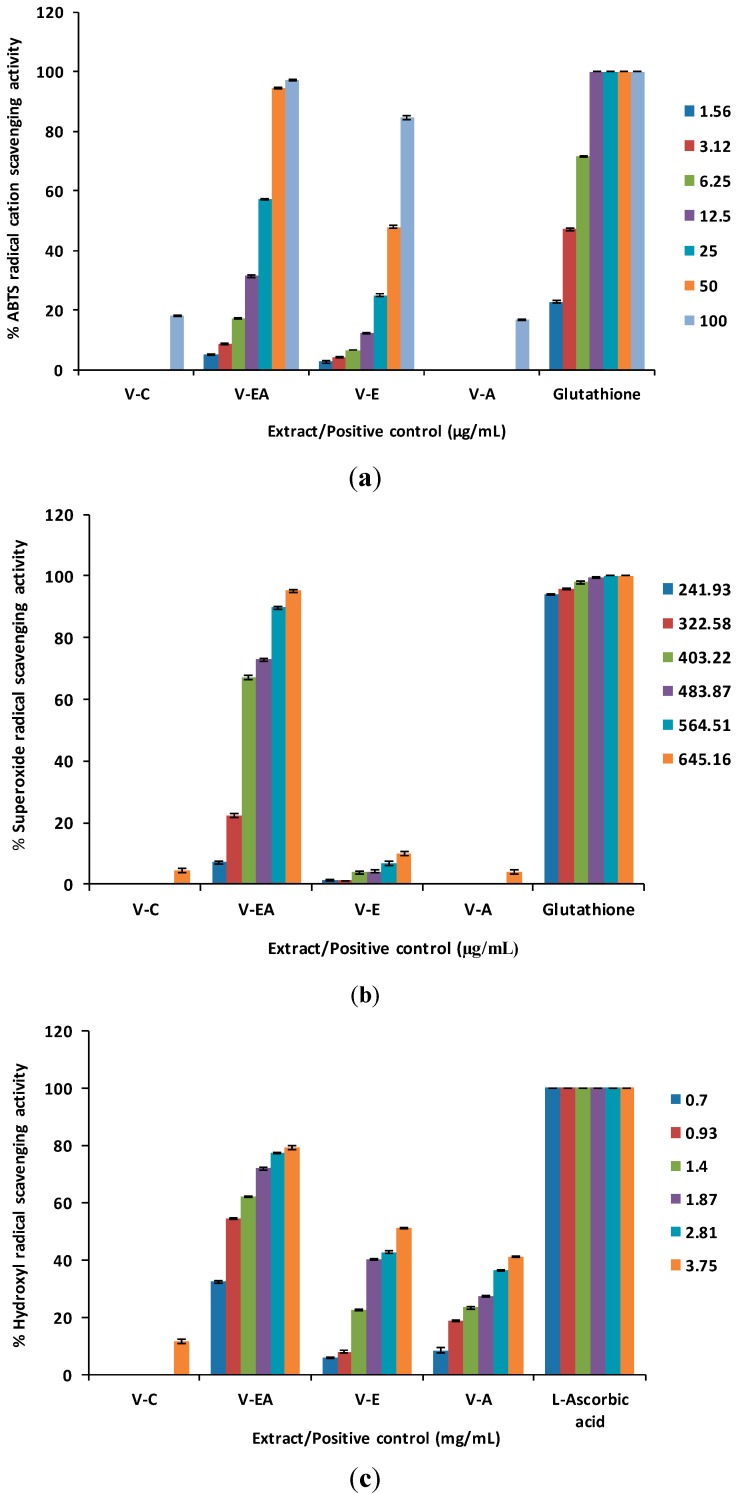

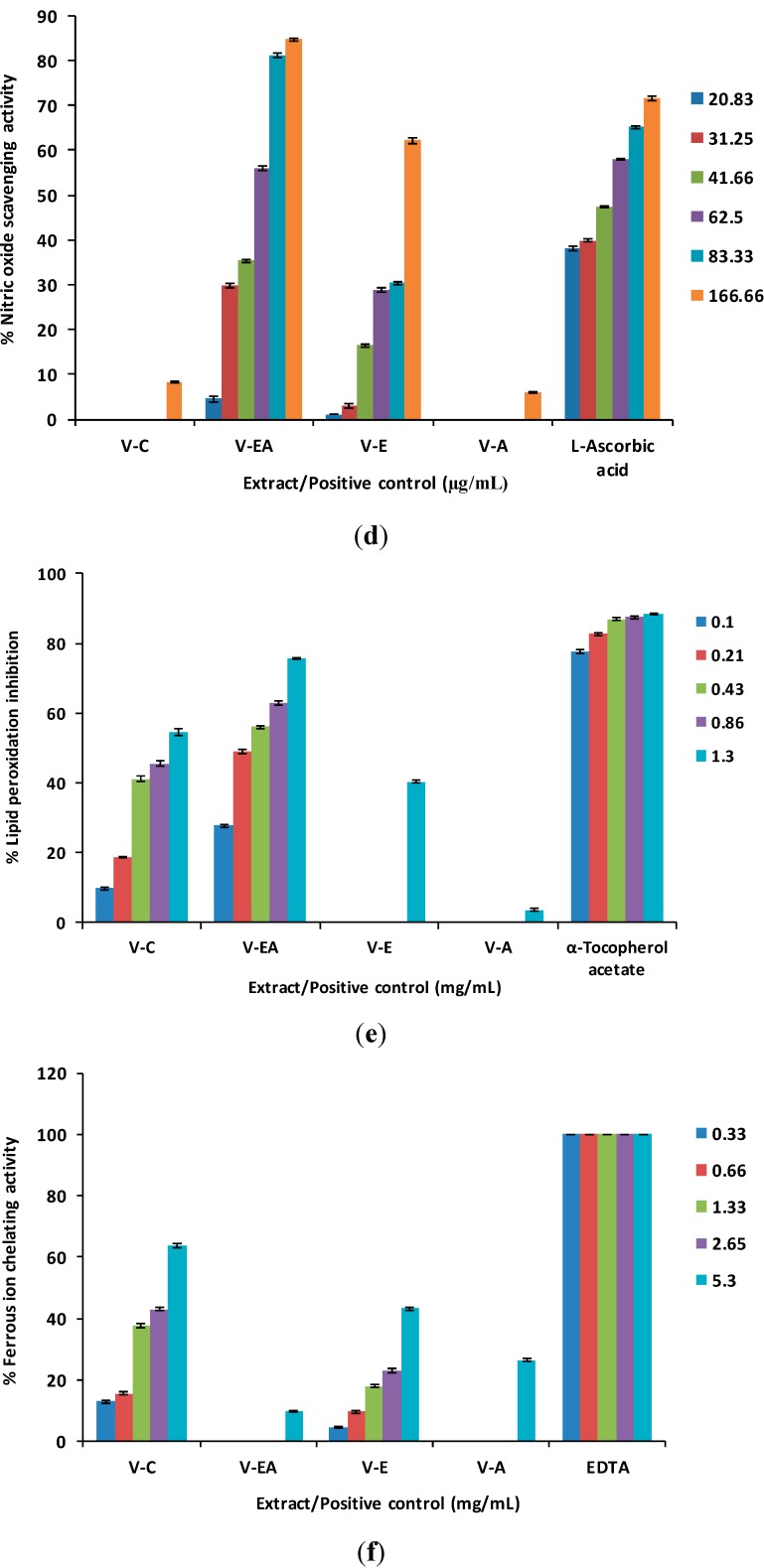

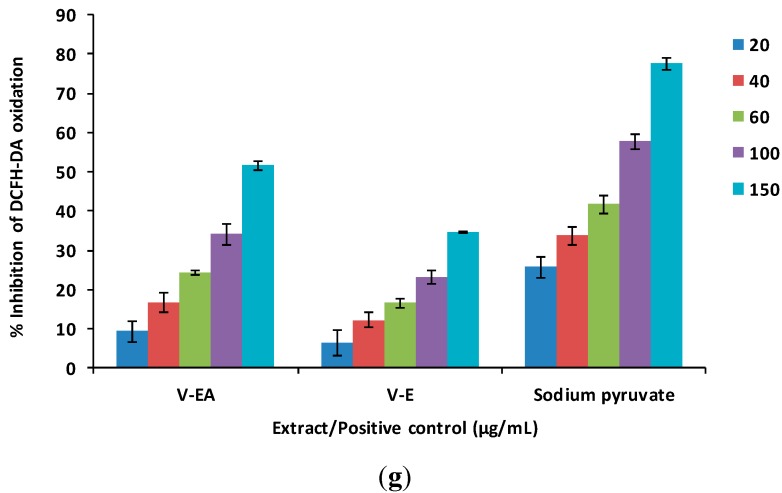
(**a**) ABTS radical cation scavenging activity. (**b**) Superoxide anion radical scavenging activity. (**c**) Hydroxyl radical scavenging activity. (**d**) Nitric oxide scavenging activity. (**e**) Lipid peroxidation inhibitory activity. (**f**) Ferrous ion chelating activity. (**g**) Antioxidant activity in MCF-12F cells.

The free radical scavenging effects of *V. kotschyana* extracts were initially evaluated against the synthetic nitrogen-centered 2,2'-azinobis(3-ethylbenzothiazoline-6-sulfonic acid (ABTS) radical cation [23]. At the highest concentration (100 µg/mL), ethyl acetate and ethanol extracts scavenged 97.30% ± 0.18% and 84.65% ± 0.56% of the radical, respectively. At the same concentration, chloroform and aqueous extracts showed weak scavenging effects (18.07% ± 0.24% and 16.95% ± 0.20%, respectively) while glutathione, the positive control, completely scavenged the radical (Figure 1a). According to the EC_50_ values, ethyl acetate extract (20.59 ± 0.07 µg/mL) was the most active; glutathione scavenged the radical with an EC_50_ value of 3.25 ± 0.02 µg/mL (Table 1). The ABTS scavenging activity of ethyl acetate extract was comparable to or better than that reported for different extracts of *V. cinerea* whole plant (EC_50_ = 19.54–26.81 µg/mL) but inferior to that of ethanol extract of *V. cinerea* leaves (EC_50_ = 12 µg/mL) [24,25]. There are also reports on extracts from other species (*Cedrus brevifolia*, *Angelica sinensis*) having higher ABTS scavenging potential (EC_50_ = 2.3–11.9 µg/mL) [26,27]. As glutathione is a powerful antioxidant, it is obvious that ethyl acetate extract, approximately six times less potent, is an efficient ABTS radical scavenger.

Further, the scavenging effects against superoxide, hydroxyl and nitric oxide radicals were evaluated [28,29,30,31]. Superoxide anion radical, generated by the autoxidation of pyrogallol [28,29], was efficiently scavenged only by the ethyl acetate extract. The superoxide scavenging activity of ethyl acetate extract increased dose-dependently from 7.11% ± 0.52% at 241.93 µg/mL to 95.15% ± 0.33% at 645.16 µg/mL. At this concentration, chloroform, ethanol and aqueous extracts exhibited only 4.56% ± 0.58%, 10.01% ± 0.60% and 4.12% ± 0.71% scavenging activity, respectively.

**Table 1:** EC_50_ values of *Vernonia kotschyana* root extracts in different antioxidant assays.

Extract/Positive Control	ABTS Radical Cation Scavenging Activity *	Superoxide Anion Radical Scavenging Activity *	Hydroxyl Radical Scavenging Activity **	Nitric Oxide Scavenging Activity *	Lipid Peroxidation Inhibitory Activity *^,^**	Ferrous Ion Chelating Activity *^,^**	Antioxidant Activity in MCF-12F Cells *
V-C	n.d.	n.d.	n.d.	n.d.	1.06 ± 0.02 **^,c,g^	3.33 ± 0.03 **^,i^	n.t.
V-EA	20.59 ± 0.07 ^a,b^	370.42 ± 0.67 ^b^	0.88 ± 0.00 ^a,d^	55.50 ± 0.27 ^a,e^	0.24 ± 0.00 **^,g,h^	n.d.	144.46 ± 2.57 ^j^
V-E	51.96 ± 0.31 ^b,c^	n.d.	3.61 ± 0.01 ^e,c^	127.63 ± 0.84^e,c^	n.d.	n.d.	n.d.
V-A	n.d.	n.d.	n.d.	n.d.	n.d.	n.d.	n.t.
Glutathione	3.25 ± 0.02 ^c,a^	31.21 ± 0.07 ^c^	n.t.	n.d.	n.t.	n.t.	n.t.
L-Ascorbic acid	n.t.	n.t.	0.11 ± 0.00 ^f,a^	46.00 ± 0.24 ^c,a^	n.t.	n.t.	n.t.
DL-α-Tocopherol acetate	n.t.	n.t.	n.t.	n.t.	17.4 ± 0.0 *^,h,c^	n.t.	n.t.
EDTA	n.t.	n.t.	n.t.	n.t.	n.t.	6.18 ± 0.08 *^,h^	n.t.
Sodium pyruvate	n.t.	n.t.	n.t.	n.t.	n.t.	n.t.	78.43 ± 3.03 ^c^

Notes: * µg/mL; ** mg/mL; n.d.-not determined due to low activity; n.t.-not tested; ^a^ *P* < 0.001* vs.* V-E; ^b^ *P* < 0.001* vs.* glutathione; ^c^ *P* < 0.001* vs.* V-EA; ^d^ *P* non-significant* vs.* L-ascorbic acid; ^e^ *P* < 0.001* vs.* L-ascorbic acid; ^f^ *P* non-significant* vs.* V-EA; ^g^ *P* < 0.001* vs.* DL-α-tocopherol acetate; ^h^ *P* < 0.001* vs.* V-C; ^i^ *P* < 0.001* vs.* EDTA; ^j^ *P* < 0.001* vs.* sodium pyruvate.

In the same concentration range (241.93–645.16 µg/mL), the scavenging effects of glutathione varied between 93.98% ± 0.16% and 101.01% ± 0.17% (Figure 1b). Hydroxyl radical, generated by Fenton reaction [30], was scavenged with different potencies by *V. kotschyana* extracts. At 3.75 mg/mL, the scavenging percentages of chloroform, ethyl acetate, ethanol and aqueous extracts were 11.70% ± 0.88%, 79.42% ± 0.68%, 51.05% ± 0.23% and 41.06% ± 0.29%, respectively. The positive control, L-ascorbic acid, scavenged hydroxyl radical more efficiently than *V. kotschyana* extracts; it completely scavenged the radical in the concentration range of 0.7 to 3.75 mg/mL (Figure 1c). Nitric oxide was generated by the decomposition of sodium nitroprusside and measured on the basis of Griess reaction [31]. The nitric oxide scavenging activity of ethyl acetate and ethanol extracts increased from 4.50% ± 0.59% and 1.15% ± 0.01%, respectively (at 20.83 µg/mL) to 84.71% ± 0.34% and 62.25% ± 0.63%, respectively (at 166.66 µg/mL). Within the same concentration range, the scavenging activity of L-ascorbic acid increased from 38.01% ± 0.46% to 71.62% ± 0.40%. At 166.66 µg/mL, chloroform and aqueous extracts showed weak scavenging activity on nitric oxide (8.37% ± 0.24% and 5.96% ± 0.27%, respectively) (Figure 1d). Among *V. kotschyana* extracts, ethyl acetate extract exhibited the strongest scavenging effects against superoxide, hydroxyl and nitric oxide radicals (EC_50_ = 370.42 ± 0.67 µg/mL, 0.88 ± 0.00 mg/mL and 55.50 ± 0.27 µg/mL, respectively). It is worthy to note its excellent nitric oxide scavenging capacity in comparison to that of the positive control, L-ascorbic acid (EC_50_ = 46.00 ± 0.24 µg/mL) (Table 1). Because of the different experimental protocols for evaluating superoxide and hydroxyl radicals scavenging effects, in many cases, a comparison of our results with other studies is not possible. Ethyl acetate extract seems to be a good scavenger of superoxide (EC_50_ = 370.42 ± 0.67 µg/mL) and hydroxyl radicals (EC_50_ = 0.88 ± 0.00 mg/mL) as other plant extracts tested in similar experimental conditions showed EC_50_ values between 0.10–2.77 mg/mL and 0.47–1.07 mg/mL, respectively [27,32,33,34]. Literature reports even lower activities for an extract and fractions from *Tuber indicum* (EC_50_ = 3.31–25.6 mg/mL) in a slightly modified hydroxyl radical scavenging assay [35]. A comparison with literature data confirmed the high nitric oxide scavenging capacity of ethyl acetate extract. At 200 µg/mL, a phenolic extract of *Pinus massoniana* bark exhibited 68.9% scavenging activity [36] while ethyl acetate extract showed a higher activity (84.71%) in a lower concentration (166.66 µg/mL). In the same experimental conditions, extracts of *Pinus brutia* bark (EC_50_ = 160.23–219.26 µg/mL) were less active than ethyl acetate extract (EC_50_ = 55.50 ± 0.27 µg/mL) [32].

### 2.2. Lipid Peroxidation Inhibitory Activity

In this assay [37], ethyl acetate extract reduced the iron-ascorbate induced lipid peroxidation of linoleic acid. At 1.3 mg/mL, the lipid peroxidation inhibitory effects of *V. kotschyana* extracts decreased as follows: ethyl acetate extract (75.66% ± 0.36%) > chloroform extract (54.52% ± 0.96%) > ethanol extract (40.27% ± 0.40%) > aqueous extract (3.73% ± 0.29%) (Figure 1e). The EC_50_ values revealed that ethyl acetate extract possessed the highest lipid peroxidation inhibitory activity (EC_50_ = 0.24 ± 0.00 mg/mL). According to the EC_50_ values, the lipid peroxidation inhibitory effect of ethyl acetate extract was less pronounced than that of DL-α-tocopherol acetate (EC_50_ = 17.4 ± 0.0 µg/mL) (Table 1) and other plant extracts that were tested using similar experimental protocols. Extracts of the roots of *Astragalus membranaceus*, *Morus alba*, *Polygonatum odoratum* and leaves of *Houttuynia cordata* and *Saururus chinensis* inhibited linoleic acid lipid peroxidation with EC_50_ values ranging from 6.2 to 15.3 µg/mL [37]. At 100 µg/mL, extracts from the aerial parts of *Galeopsis* *speciosa*, *Lamium purpureum*, *Lamium album*, *Leonurus cardiaca*, *Stachys officinalis* and *Marrubium* *vulgare* reduced lipid peroxidation by 64.5%–78.7% [38]; for comparison, at the same concentration, ethyl acetate extract exhibited 27.72% activity.

### 2.3. Ferrous Ion Chelating Activity

In the ferrous ion chelating assay [39,40], the chloroform extract proved to be the most active. At 5.3 mg/mL, its ferrous ion chelating ability reached 63.83% ± 0.87%. At the same concentration, the ethanol, aqueous and ethyl acetate extracts exhibited only 43.31% ± 0.52%, 26.52% ± 0.60% and 9.81% ± 0.32% chelating activity, respectively (Figure 1f). According to the EC_50_ values, the chelating ability of chloroform extract was lower than that of EDTA (3.33 ± 0.03 mg/mL* vs.* 6.16 ± 0.08 µg/mL) (Table 1). Tested under the same experimental conditions, extracts of *Acacia confusa* bark (EC_50_ = 253–2185.6 µg/mL) and *Pinus cembra* needles (EC_50_ = 1755 µg/mL) showed stronger chelating effects than chloroform extract [40,41].

### 2.4. Antioxidant Activity in MCF-12F Cells

Ethyl acetate and ethanol extracts showed the highest activity in free radical scavenging and lipid peroxidation inhibition assays. Therefore, these extracts were assessed for their ability to reduce dichlorodihydrofluorescein diacetate (DCFH-DA) oxidation in MCF-12F cells exposed to exogenous hydrogen peroxide [42]. At 150 μg/mL, the ethyl acetate extract decreased DCFH-DA oxidation by 51.56% ± 1.17%, while the ethanol extract reduced it only by 34.51% ± 0.20% (Figure 1g). Ethyl acetate extract (EC_50_ = 144.46 ± 2.57 μg/mL) was more active than ethanol extract (EC_50_ > 150 μg/mL) but less active than sodium pyruvate, the positive control (EC_50_ = 78.43 ± 3.03 μg/mL) (Table 1).

### 2.5. Phytochemical Screening

The phytochemical study revealed the presence of steroidal compounds in all extracts. Phenols (tannins), flavonoids and saponins were detected in ethyl acetate and ethanol extracts. Additionaly, free sugars were identified in the ethanol extract. Aminoacids/proteins, polysaccharides and saponins were found to be present in aqueous extract [43,44].

### 2.6. FT-IR Spectroscopic Analysis

**Figure 2:**
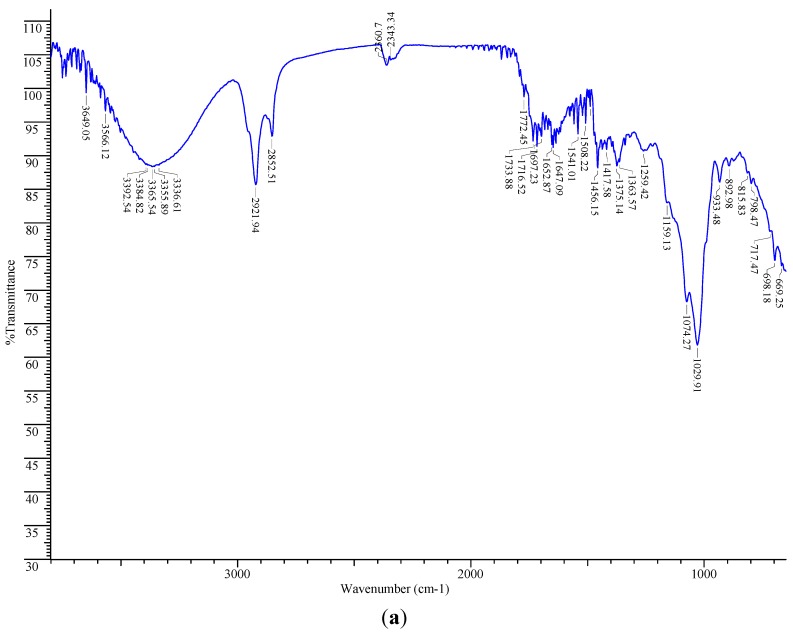

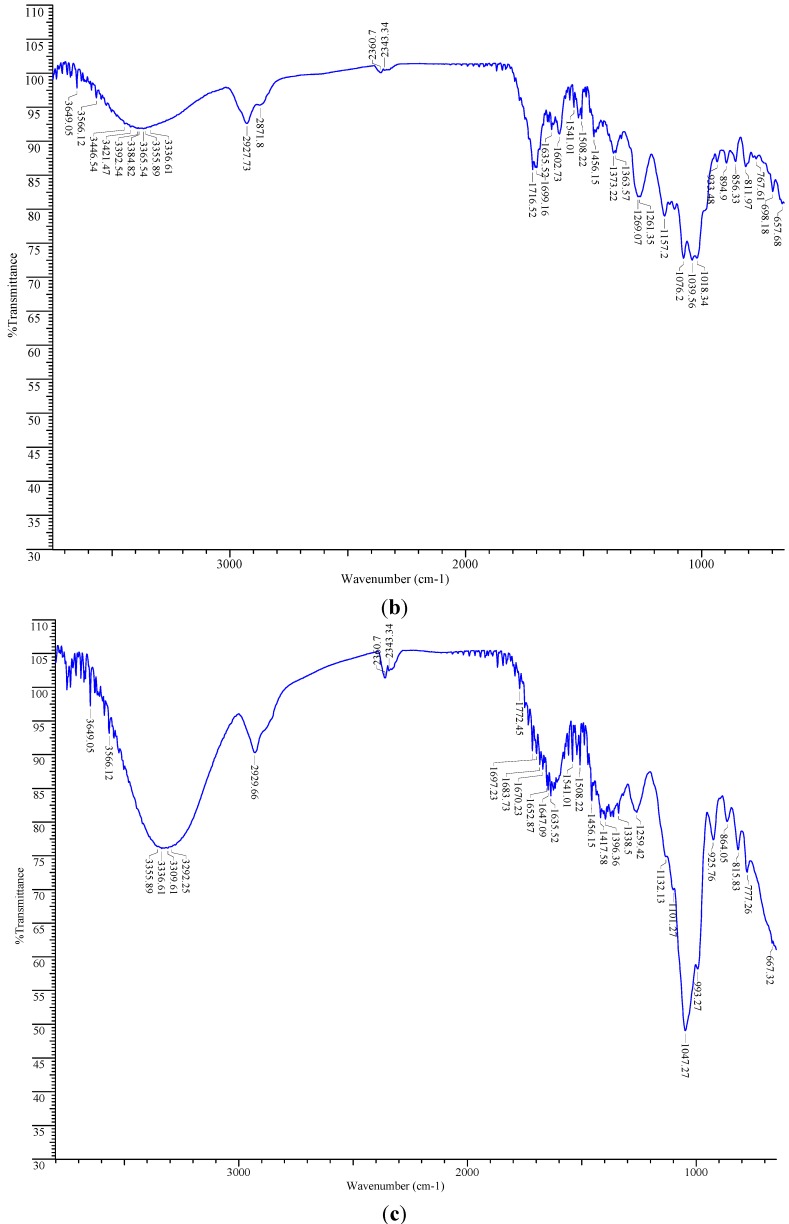

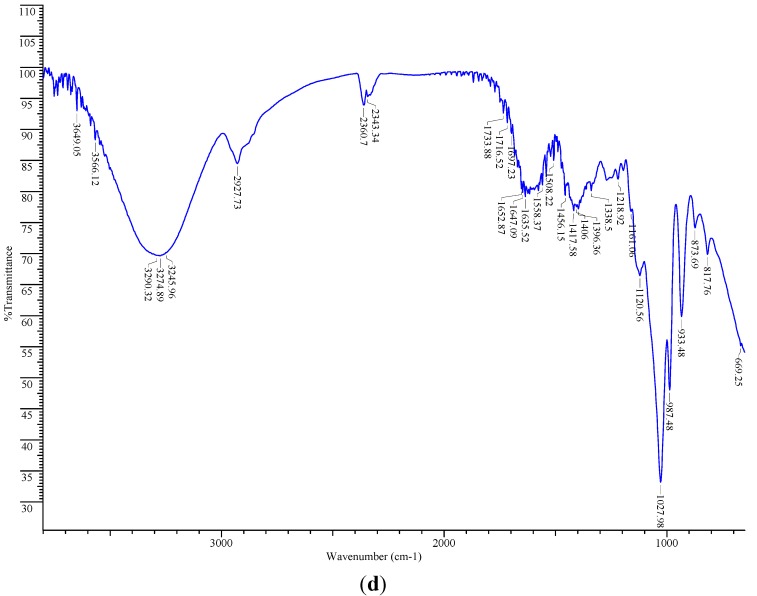
(**a**) FT-IR spectra of chloroform extract. (**b**) FT-IR spectra of ethyl acetate extract. (**c**) FT-IR spectra of ethanol extract. (**d**) FT-IR spectra of aqueous extract.

*V. kotschyana* extracts were analysed by FT-IR spectroscopy. Chloroform extract showed strong absorption bands belonging to the stretching vibration of O-H (3649–3336 cm^−1^) and saturated hydrocarbon groups (2921 cm^−1^, 2852 cm^−1^). The bands at 1159 cm^−1^, 1074 cm^−1^ and 1029 cm^−1^ belong to the stretching vibration of C-O and bending vibration of C-O-H groups. Other bands could be assigned to the stretching vibration of C=O groups and aliphatic CC double bonds (1716–1647 cm^−1^) and bending vibration of methyl/methylene groups (1456–1363 cm^−1^) (Figure 2a). These spectral data indicate that long-chain alkyl (strong bands around 2920 cm^−1^ and 2850 cm^−1^) and steroidal compounds (bands at 3650–3590 cm^−1^ for free O-H, 2960–2850 cm^−1^ for C-H stretching, 1485–1445 cm^−1^ for CH_2_ bending) are present in chloroform extract [45,46]. Absorption bands which could be assigned to steroidal compounds were present in all extracts.

In ethyl acetate extract, there were also present bands corresponding to the stretching vibration of O-H (3649–3336 cm^−1^) and saturated hydrocarbon groups (2927 cm^−1^, 2871 cm^−1^), stretching vibration of C-O and bending vibration of C-O-H groups (1157–1018 cm^−1^), stretching vibration of C=O groups and aliphatic CC double bonds (1716 cm^−1^, 1699 cm^−1^, 1635 cm^−1^). The bending vibration of methyl/methylene groups appeared at 1456–1363 cm^−1^. In addition, bands corresponding to skeletal stretching vibration of the aromatic rings and =C-O-C group of flavonoids (1602 cm^−1^, 1508 cm^−1^, 1456 cm^−1^, 1269 cm^−1^, 1261 cm^−1^) were visible [47] (Figure 2b). Weaker absorption bands for phenolic compounds were also present in chloroform, ethanol and aqueous extracts (1508 cm^−1^, 1456 cm^−1^, 1259 cm^−1^). These spectral data are in agreement with the quantitative study that showed the highest phenolic content in ethyl acetate extract followed by ethanol, chloroform and aqueous extracts (Table 2).

**Table 2:** Phenolic content of *Vernonia kotschyana* root extracts.

Extract	Total Phenolic Content (g GAE/100 g)
V-C	1.08 ± 0.03 ^a,b,c^
V-EA	14.89 ± 0.02 ^b,c,d^
V-E	5.99 ± 0.07 ^c,d,a^
V-A	0.78 ± 0.01 ^d,a,b^

Notes: ^a^ *P* < 0.001* vs.* V-EA, ^b^ *P* < 0.001* vs.* V-E, ^c^ *P* < 0.001* vs.* V-A, ^d^ *P* < 0.001* vs.* V-C.

The spectra of ethanol and aqueous extracts were rather similar showing strong absorption bands attributed to the stretching vibrations of O-H (3649–3292 cm^−1^ and 3649–3245 cm^−1^, respectively) and saturated hydrocarbon groups (2929 cm^−1^ and 2927 cm^−1^, respectively), stretching vibration of C-O and bending vibration of C-O-H groups (1132–993 cm^−1^ and 1218–987 cm^−1^, respectively), stretching vibration of C=O groups and aliphatic CC double bonds (1716–1635 cm^−1^). Bands corresponding to the bending vibration of methyl/methylene groups appeared in both spectra at 1456–1338 cm^−1^ (Figure 2c,d). In ethanol and aqueous extracts, the bands corresponding to the stretching vibration of C-O and bending vibration of C-O-H groups (1132–993 cm^−1^ and 1218–987 cm^−1^, respectively) were stronger as compared to chloroform and ethyl acetate extracts (1159–1029 cm^−1^ and 1157–1018 cm^−1^, respectively) indicating high amounts of carbohydrates (mono-, oligosaccharides in ethanol extract and polysaccharides in aqueous extract) [48]. In case of aqueous extract, bands at 3400–3200 cm^−1^, 1700–1600 cm^−1^ and 1575–1480 cm^−1^ could also be assigned to proteins/aminoacids (NH stretching, C=O stretching and in-plane NH bending/CN stretching vibrations, respectively) [49,50] (Figure 2d).

These spectral data are consistent with previous studies reporting that *V. kotschyana* roots contain steroidal glycosides of stigmastane and androstane type (the former are known as vernoniosides) and carbohydrates (pectic polysaccharides, inulin type fructans) [18,19,51]. A FT-IR analysis of *V. cinerea* reported the presence of aminoacids, alkenes, ethers, organic halogen compounds and carbohydrates in the whole plant [52].

### 2.7. Total Phenolic Content

Ethyl acetate extract had the highest total phenolic content (14.89% ± 0.02%), followed by ethanol extract (5.99% ± 0.07%). The lowest amounts of polyphenols were detected in chloroform and aqueous extracts (1.08% ± 0.03% and 0.78% ± 0.01%, respectively) (Table 2).

There are reports on phenolic contents in extracts obtained from other *Vernonia* species: 0.147% in the methanol extract of *V. cinerea* whole plant, 0.19%–23.11% in the ethanol extract of *V. condensata* leaves and its fractions, 0.35% in the aqueous extract of *V. amygdalina* leaves [25,53,54]. In light of these literature data, ethyl acetate extract of *V.* *kotschyana* roots contains significant amounts of polyphenols.

Among all extracts, the ethyl acetate extract exhibited the most promising antioxidant effects. It was the most active in free radical scavenging and lipid peroxidation inhibition assays but it showed a very weak activity in ferrous ion chelating assay. Free radical scavenging involves either hydrogen donation or electron transfer [55]. Ferrous ion chelation is another important antioxidant mechanism. Ferrous ions have pro-oxidant effects; they promote the generation of the highly reactive hydroxyl radical via Fenton reaction [56]. The results of this study clearly show that the antioxidant activity of the ethyl acetate extract is mainly based on its reducing capacity (hydrogen donation/electron transfer) without involving chelation of ferrous ions. The results of the chemical studies corroborated with those of the antioxidant assays suggest that polyphenols are mainly responsible for the antioxidant effects of ethyl acetate extract; other compounds than phenolics confer ferrous ion chelating capacity to chloroform extract. However, antiulcer activity of many plant extracts has been attributed to their antioxidant constituents, especially polyphenols. Gallic acid is mainly responsible for the protective activity of the aqueous extract of *Zingiber officinale* rhizome against oxidative stress-induced gastric mucosal damage [10]. Phytochemical studies revealed high contents of total phenolics and flavonoids (159.76 ± 0.32 mg/g and 79.51 ± 0.57 mg/g, respectively) besides polysaccharides (506.7 ± 5.2 mg/g) in *Opuntia ficus indica f. inermis* flowers extract; the extract exhibited remarkable* in vivo* antiulcerogenic effects [13]. Anthocyanins are mainly responsible for the gastroprotective activity of strawberry extracts. They have high ROS scavenging effects; in addition, they enhance the biosynthesis of mucopolysaccharides and consequently the protective activity of the mucus layer. Pretreatment with cyanidin-3-glucoside, a very common anthocyanoside, significantly reversed ethanol-induced oxidative stress in gastric mucosa (increased lipid peroxidation, depletion of glutathione level and activities of antioxidant enzymes such as superoxide dismutase, catalase, glutathione peroxidase) [14]. Gastroprotective effects of *Curcuma longa* and *Mentha piperita* aqueous extracts were also attributed to polyphenols, mainly flavonoids [15]. Besides their significant ROS scavenging effects, flavonoids increase prostaglandin secretion and decrease histamine secretion from mast cells [14].

No matter the causative agent (*Helicobacter pylori*, alcohol, stress, NSAIDS), gastric ulceration is characterized by an increase in oxidative stress. The ethyl acetate extract showed the most potent antioxidant effects in* in vitro* studies, making it a good candidate for* in vivo* evaluation of a possible antiulcer activity in relation to its antioxidant potential. In addition, other gastroprotective mechanisms such as antisecretory and cytoprotective effects should be investigated as polyphenols have been reported to exhibit such effects in the gastrointestinal tract [57,58].

It is worthy to note that superoxide, hydroxyl, nitric oxide radicals, hydrogen peroxide and lipid peroxidation play a critical role in many other oxidative-stress related pathological conditions and diseases. Superoxide anion radical and other ROS species derived from it (hydrogen peroxide, hydroxyl radical) are responsible for myocardial tissue damage in ischemia/reperfusion injury; they are also involved in the pathogenesis of hypertension, chronic obstructive pulmonary disease, diabetic complications (coronary and peripheral artery diseases, diabetic retinopathy, nephropathy and neuropathy) [59,60]. Hydroxyl radical-induced DNA damage is involved in the initiation, promotion and progression of carcinogenesis [61]. High levels of nitric oxide produced by iNOS have been detected in inflammatory disorders of the joints, lungs and gut, atherosclerosis, cancer and diabetes [62]. Lipid peroxidation is crucial in the development of atherosclerosis and several neurodegenerative diseases (amyotrophic lateral sclerosis, Alzheimer's and Parkinson's diseases) [63,64]. All these facts suggest a possible use of the ethyl acetate extract as ingredient in dietary supplements for pathological conditions and diseases associated with oxidative stress. *In vivo* studies on antiulcer and antioxidant potential of ethyl acetate extract are in progress.

## 3. Experimental Section

### 3.1. Chemicals

Tris(hydroxymethyl)aminomethane (Tris), hydrochloric acid, ferrous chloride, sodium chloride, sodium nitroprusside were purchased from Merck (Darmstadt, Germany). ABTS diammonium salt, ethylenediaminetetraacetic acid (EDTA), 3-(2-pyridyl)-5,6-diphenyl-1,2,4-triazine-4',4"-disulfonic acid monosodium salt (ferrozine), disodium phosphate dodecahydrate, monopotassium phosphate, glutathione, pyrogallol, Folin-Ciocalteu’s phenol reagent were supplied by Fluka (Steinheim, Germany). DL-α-Tocopherol acetate was from Sigma-Aldrich (Buchs, Switzerland). Sodium carbonate, iron (II) sulfate heptahydrate, hydrogen peroxide, L-ascorbic acid, linoleic acid, thiobarbituric acid, *N*-(1-naphthyl)ethylenediamine dihydrochloride, sodium salicylate, sulfanilamide, acetic acid were obtained from Sigma-Aldrich Chemie GmbH (Steinheim, Germany). Gallic acid monohydrate, trichloroacetic acid and potassium persulfate were purchased from Riedel-de Haën (Seelze, Germany). Dulbecco’s Modified Eagle’s Medium/Ham’s Nutrient Mixture F-12 (DMEM/F-12), horse serum, antibiotic-antimycotic and trypsin were purchased from Gibco/Invitrogen (Carlsbad, CA, USA). Insulin, hydrocortisone, cholera toxin, epidermal growth factor (EGF), DCFH-DA, sodium pyruvate were purchased from Sigma (St. Louis, MO, USA).

### 3.2. Cell Culture

MCF-12F cell line (normal human mammary epithelial cell line) was obtained from the American Type Culture Collection (ATCC, Manassas, VA, USA). MCF-12F cells were routinely cultured in DMEM/F-12 supplemented with 5% horse serum, 1% antibiotic-antimycotic, 10 μg/mL insulin, 500 ng/mL hydrocortisone, 100 ng/mL cholera toxin and 20 ng/mL EGF. Cells were grown in a humidified atmosphere with 5% CO_2_ at 37 °C.

### 3.3. Plant Material

The roots of *Vernonia kotschyana* were kindly supplied by the Department of Traditional Medicine, Bamako, Mali. The identity of plant material was authenticated by Prof. Drissa Diallo, Director of the Department of Traditional Medicine in Bamako. A voucher specimen (VKR2011) is deposited in the Laboratory of Pharmacognosy, Faculty of Pharmacy, *Grigore T. Popa* University of Medicine and Pharmacy — Iasi, Romania.

### 3.4. Preparation of Extracts

Dried and powdered roots (200 g) were extracted successively with chloroform (3 × 1000 mL, each time for 4 h) under reflux in a water bath at 50 °C. The vegetal residue was extracted sequentially with ethyl acetate, ethanol and water as described above. The chloroform, ethyl acetate and ethanol extracts were evaporated to dryness under reduced pressure at 40 °C while the water extract was lyophilized. All extracts were stored at 4 °C until use.

### 3.5. ABTS Radical Cation Scavenging Assay

The assay was performed as described by Re* et al.*, [23]. ABTS radical cation, generated by incubation of ABTS (7 mM) with potassium persulfate (2.45 mM) for 12–16 h in dark, was further diluted with ethanol to an absorbance of 0.70 ± 0.02 at 734 nm. In order to evaluate the scavenging activity, extracts (0.15–10 mg/mL) were mixed with ABTS radical cation solution in a total volume of 2 mL. The decay in absorbance at 734 nm was measured after 6 min of reaction at 30 °C. Glutathione was used as positive control. Percent ABTS radical cation scavenging activity was calculated using the following formula: 100 × (A_control_ − A_sample_/A_control_) where A_control_ is the absorbance of the control and A_sample_ is the absorbance in the presence of extracts/glutathione.

### 3.6. Superoxide Anion Radical Scavenging Assay

A slightly modified method of Marklund and Marklund was used to evaluate superoxide scavenging effects [28,29]. The reaction mixture (3.1 mL) contained different concentrations of extracts (7.5–20 mg/mL), 1 mM EDTA in 50 mM Tris-HCl buffer at pH 8.0 and 6 mM pyrogallol. The increase in absorbance at 325 nm was measured every 30 s for 4 min. Glutathione was the positive control. Percent superoxide anion radical scavenging activity was calculated by the formula: 100 × (slope_control_ − slope_sample_/slope_control_) where slope_control_ and slope_sample_ are the slopes of the plots of absorbance* vs.* time for control and samples, respectively [28].

### 3.7. Hydroxyl Radical Scavenging Assay

The assay was performed as reported by Jeong* et al.* [30]. The reaction mixture, containing extracts (5–40 mg/mL), 1.5 mM iron (II) sulfate heptahydrate, 20 mM sodium salicylate and 6 mM hydrogen peroxide in a total volume of 2.4 mL, was incubated at 37 °C for 30 min. After cooling, the absorbance was measured at 562 nm. L-Ascorbic acid was used as positive control. Percent hydroxyl radical scavenging activity was calculated using the formula: 100 × (A_control_ − A_sample_/A_control_).

### 3.8. Nitric Oxide Scavenging Assay

Nitric oxide scavenging activity was evaluated according to the method described by Tsai* et al.* [31]. Extracts (0.62–5 mg/mL) were mixed with 10 mM sodium nitroprusside followed by dilution with phosphate buffered saline to give a final volume of 3 mL. After 150 min incubation at 25 °C, an aliquot of the reaction mixture (0.5 mL) was mixed with 0.33% sulfanilamide in 20% acetic acid (1 mL) and 0.1% naphthylethylenediamine dihydrochloride (1 mL). After 30 min the absorbance was measured at 540 nm. L-Ascorbic acid was the positive control. Percent nitric oxide scavenging activity was calculated as 100 × (A_control_ − A_sample_/A_control_).

### 3.9. Lipid Peroxidation Inhibition Assay

Inhibition of lipid peroxidation was assessed by the method described by Choi* et al.* with minor changes [37]. In brief, the reaction mixture, containing extracts (2.5–30 mg/mL), 20 mM linoleic acid, 4 mM iron (II) sulfate heptahydrate, 2 mM ascorbic acid and 100 mM Tris buffer (pH 7.5) in a total volume of 1.3 mL, was incubated at 37 °C for 30 min. A volume of 1 mL of 5.5% trichloroacetic acid and 1% thiobarbituric acid was further added followed by heating at 95 °C for 20 min. After cooling and centrifugation, the absorbance was measured at 532 nm. dl-α-Tocopherol acetate was the positive control. Percent lipid peroxidation inhibition was calculated using the following formula: 100 × (A_control_ − A_sample_)/(A_control_ − A_blank_) where A_blank_ is the absorbance of the mixture containing only linoleic acid and Tris buffer.

### 3.10. Ferrous Ion Chelating Assay

The assay was carried out according to the method of Dinis* et al.*, with minor changes [39,40]. Extracts (2.5–40 mg/mL) were mixed vigorously with 2 mM ferrous chloride, 5 mM ferrozine and ethanol in a total volume of 3.02 mL. After 10 min incubation at room temperature, the absorbance was determined at 562 nm. EDTA was used as positive control. Percent ferrous ion chelating activity was calculated as follows: 100 × (A_control_ − A_sample_/A_control_).

### 3.11. Cell-Based Antioxidant Assay

Antioxidant activity in MCF-12F cells was evaluated according to the method reported by Girard-Lalancette* et al.* with some modifications [42]. Hydrogen peroxide was used as intracellular oxidizing agent instead of *tert*-butylhydroperoxide. Briefly, MCF-12F cells were seeded in transparent 12-well plates at a final concentration of 4 × 10^4^ cells/well and incubated at 37 °C in 5% CO_2_ humidified atmosphere. After 24 h, the medium was removed and the cells were further treated with 1 mL of DMEM/F-12 containing 100 μM DCFH-DA followed by addition of 1 μL of extracts or sodium pyruvate (20–150 mg/mL) and 1.1 μL of 100 μM hydrogen peroxide. Inside the cells, DCFH-DA is hydrolysed to DCFH by intracellular esterases; in the presence of ROS, DCFH is oxidized to highly fluorescent DCF. DCF fluorescence intensity was measured after 30 min incubation with hydrogen peroxide using an Infinite F200 PRO multimode reader (Tecan, Männedorf, Switzerland) (excitation wavelength 485 nm, emission wavelength 535 nm).

### 3.12. Phytochemical Screening

Identification of the main constituents was performed according to standard procedures: Liebermann’s and Salkowski’s tests for steroidal ring, foam test for saponins, ferric chloride test for phenols (tannins), sodium hydroxide test for flavonoids, Fehling's test for free sugars and polysaccharides (for the latter, after a previous acidic hydrolysis) and ninhydrin test for aminoacids/proteins [43,44].

### 3.13. FT-IR Spectroscopic Analysis

FT-IR spectra were recorded on ABB MB3000 FT-IR spectrometer (Québec, QC, Canada). FT-IR spectra were acquired over a range of 4000–650 cm^−1^ with 16 scans at a spectrum resolution of 4 cm^−1^. Spectra processing was carried out using the Horizon MB software.

### 3.14. Quantification of Total Phenolic Content

Total phenolic content was quantified by Folin-Ciocalteu method as previously described [65]. In brief, a reaction mixture (4 mL) containing extracts, Folin-Ciocalteu’s phenol reagent and 20% sodium carbonate solution was incubated for 2 h in dark at room temperature. Absorbance was measured at 765 nm. The results were expressed as g of gallic acid equivalents (GAE) *per* 100 g of extract.

### 3.15. Statistical Analysis

All experiments were performed in triplicate and the results were expressed as mean ± standard error. The EC_50_ values were calculated by linear interpolation between values above and below 50% activity. Statistical evaluation was performed using Student’s *t* test; *P* < 0.05 was considered statistically significant.

## 4. Conclusions

*Vernonia kotschyana* roots have been used in Malian traditional medicine for the treatment of gastroduodenal ulcers and gastritis. To the best of our knowledge, this is the first report on the antioxidant activity of *Vernonia kotschyana* roots. Our study revealed that an ethyl acetate extract of the roots efficiently scavenged ABTS, superoxide, hydroxyl and nitric oxide radicals and inhibited lipid peroxidation. The extract also showed a high hydrogen peroxide scavenging capacity in normal cells. As superoxide, hydroxyl, nitric oxide radicals, hydrogen peroxide and lipid peroxidation are known to be involved in gastric ulceration, the ethyl acetate extract merits further investigation of its antiulcer activity. Identification of this antioxidant potential in the ethyl acetate extract may extend the use of the roots towards the prevention and treatment of other diseases characterized by an increase in oxidative stress.
